# Inflammation

**Published:** 1861-08

**Authors:** W. H. Atkinson


### DENTAL REGISTER OF THE WEST.

Vol. XV.] AUGUST, 1861. [No. 8.

### Original Essays and Communications.

### INFLAMMATION.

BY W. H. ATKINSON.

INFLAMMATION is but perverted nutrition, and hence, must be present in every case of abnormal action of any organ. That this may be demonstrably clear, it will be necessary to examine the stages, not only of abnormal, but normal nutrition.

Then to define :—the term primarily signifies to nourish. Now, as no nourishment can be appropriated to the system in a solid state, solution is the first prerequisite to effect the transfer of the entities from one state to another.

To effect solution, a certain grade of affinity must be secured between the menstruum and the entities to be dissolved, to hold them in solution and in undisturbed repose for a time, until introduced to the system, where a higher affinity is found for the entities in solution, and thus they, under the attraction of this affinity, leave the menstruum, and take up their residence in the tissue in strongest relation to them, and thus the molecule is recuperated. This is nutrition, so far as the primal, perceptible action is concerned.

The secondary action is not quite so apparent to the severely logical perception; but is another play of affinity between solutions, i. e., the spent cells, having deteriorated the circulation, render it impure, or effete, in the exact ratio of the quantity of spent cells contaminating it. Now, as soon as the nutrient solution has found its way into this circulation, in sinus, sac, artery, vein, or capillary, the inceptive act to nutrition takes place, whch inaugurates both the primary and secondary acts, that together, in their completeness, constitute nutrition.

The secondary act, alone considered, is the play of affinity between the effete matters in the circulation and the menstruum holding life entities in the nutrient pabulum, which renders it easy for these to escape into the plasma of the circulation, for the purpose of renovating the worn or weakened cells, or the formation of new ones, which completes the act of a true nutriticn or growth. The products of this quadruple affinity are invigorated tissues, or new tissues, on the one hand, and effete solutions, or debris, on the other.

Now, it will be well to reflect that the debris of one form of organic life is just the true pabulum for another ; so that, in the grand round of the circulation, opportunity for the greatest economy is provided ; and when all these affinities are in full play, all vitalized entities are taken up and used, and nothing but absolutely effete matters are thrown into the common draught, or waste pipes of the economy. Thus, at a minimum expense of food and functional activity, have we secured a perfect nutrition.

This opens a wide and extended field, in any department of which, the inceptive act of *inflammation* may exhibit its special character. But that this exhibit be plain to us, a very close scrutiny is requisite ; for it is in these inceptive stages only, that inflammation is distinct, unitary, and uncomplicated. If preserved in these stages, it is always capable of antidotal, analytic annihilation—these being the common and proper exhibitions of the true "*vis medicatrix naturæ*," to simulate which should always be the endeavor of the enlightened medical adviser.

That we may see some of the gateways into this vast field of causation, I will endeavor to point out a few of them.

1st. A disagreement between the constituents of the nutrient solution themselves may be the cause of inceptive perverted nutrition.

2d. Incongruity of an inter se complacent, or harmonious solution, with the circulation, in whole or in part, or the molecule, cell or tissue to which destined, may set up an inharmonic action, at any one of these stages, or places of metamorphosis, which will modify, to almost endless variety, this action called inflammation, which I propose to denominate "*perverted nutrition.*"

Compatibility or incompatibility of food prove the above positions. For instance, a food, perfectly compatible and health-sustaining in one state of the system, will be slightly inconvenient, irritative, or absolutely destructive (incompatible) in another state of the general chylopoietic and securing system; thus proving that the ability to appropriate is as necessary as the presentation of a proper pabulum for appropriation.

The disagreements of untried food, or change of food, is a familiar example of this want of harmony of the affininities of food and system. To many digestive organs, the ordinary salted meats are poisonous (incompatible) at first, for very obvious reasons. But, after long struggling, the majority will be enabled to overcome the disgust for the improper food, and, by a sort of compromise, reject a part, and accept a portion, and make it do; but this is not perfect health. For the sensitiveness of the organism to the noxious solution must be overcome, either by blunting or destruction. In either case, we have but a part of the functions of the system left to perform the complicated digestion. If but a single system of organs were in want of nutrition, the solution of nutriment need not be complicated. For example, if muscle alone needed recuperation, next to nothing of the phosphates need enter the solution, albumen alone being capable of sustaining this tissue, with a very little iron, added for the use of the higher organizations of this tissue.

Now that we have seen that inflammations can only occur in tissues at the point of passing from the solid to the fluid, or from the fluid to the solid state, the astute, philosophic mind will readily perceive that all we have to do is to arrest the further change in either direction. And as all inflammations are but too rapidly oxydating the inflammable constituents of organic bodies, we may see the imperative propriety of "starving the fever"—ceasing to supply fuel to the fire. And the best way to do this is to reduce the temperature of the patient to the lowest point compatible with the continuance of life, giving him nothing but pure water, externally and internally.

